# Land cover, more than monthly fire weather, drives fire-size distribution in Southern Québec forests: Implications for fire risk management

**DOI:** 10.1371/journal.pone.0179294

**Published:** 2017-06-13

**Authors:** Jean Marchal, Steve G. Cumming, Eliot J. B. McIntire

**Affiliations:** 1 Département des Sciences du Bois et de la Forêt, Pavillon Abitibi-Price, Université Laval, Québec, Québec, Canada; 2 Canadian Forest Service, Natural Resources Canada, Victoria, British Columbia, Canada; Ecole Pratique des Hautes Etudes, FRANCE

## Abstract

Fire activity in North American forests is expected to increase substantially with climate change. This would represent a growing risk to human settlements and industrial infrastructure proximal to forests, and to the forest products industry. We modelled fire size distributions in southern Québec as functions of fire weather and land cover, thus explicitly integrating some of the biotic interactions and feedbacks in a forest-wildfire system. We found that, contrary to expectations, land-cover and not fire weather was the primary driver of fire size in our study region. Fires were highly selective on fuel-type under a wide range of fire weather conditions: specifically, deciduous forest, lakes and to a lesser extent recently burned areas decreased the expected fire size in their vicinity compared to conifer forest. This has large implications for fire risk management in that fuels management could reduce fire risk over the long term. Our results imply, for example, that if 30% of a conifer-dominated landscape were converted to hardwoods, the probability of a given fire, occurring in that landscape under mean fire weather conditions, exceeding 100,000 ha would be reduced by a factor of 21. A similarly marked but slightly smaller effect size would be expected under extreme fire weather conditions. We attribute the decrease in expected fire size that occurs in recently burned areas to fuel availability limitations on fires spread. Because regenerating burned conifer stands often pass through a deciduous stage, this would also act as a negative biotic feedback whereby the occurrence of fires limits the size of nearby future for some period of time. Our parameter estimates imply that changes in vegetation flammability or fuel availability after fires would tend to counteract shifts in the fire size distribution favoring larger fires that are expected under climate warming. Ecological forecasts from models neglecting these feedbacks may markedly overestimate the consequences of climate warming on fire activity, and could be misleading. Assessments of vulnerability to climate change, and subsequent adaptation strategies, are directly dependent on integrated ecological forecasts. Thus, we stress the need to explicitly incorporate land-cover’s direct effects and feedbacks in simulation models of coupled climate–fire–fuels systems.

## Introduction

Fire is a major disturbance process structuring ecosystems and influencing the distribution of biodiversity around the globe [1,2]. Fire activity in boreal forests has been predicted to substantially increase over the 21^st^ century, in response to climate warming [3–5]. However, biotic interactions and feedbacks related to land-cover effects are rarely included in the models used to forecast fire activity. This is because, until recently [6–8], land-cover effects were believed to be negligible relative to fire weather, likely because of the lack of heterogeneity in land-cover data [9, 10]. If land-cover effects are in fact *not* negligible, then the reliability of these forecasts is questionable. This limitation of our understanding of ecosystem functioning limits our ability to reliably forecast how climate change will impact climate–fire–fuels systems. Therefore, there are non-trivial risks of developing inappropriate fire management and mitigation strategies.

A fire size distribution (FSD) is an empirical distribution, or a theoretical model, of the fire sizes characteristic of a given temporal and spatial extent. In comparison to some indicators of fire regime, such as fire frequency [10–12] or the rate of burn [4,5,9], FSDs have been relatively little studied and there are still important gaps in knowledge [13]. The distribution of fire sizes emerges from complex interactions among top-down controls of fire spread, e.g. fire weather, and bottom-up controls such as the spatial configuration of physical barriers, e.g. water bodies, and fuels [13]. A coupled fire-fuels system exhibits strong feedbacks in that past fires negatively influence the spread of subsequent fires by reducing fuel availability [14,15] and altering fuel composition [16,17]. Furthermore, there is a growing body of evidence that fires do not burn all types of fuels indiscriminately, but rather display type-specific preferences and avoidances [15,18–20]. For example, northern hardwoods are often regarded as “fireproof” during the growing season [21,22].

A variety of probability distributions have been used to model FSDs [23], the family of power-law or Pareto distributions being most often supported by data [13]. However, FSDs rarely if ever follow a pure power-law distribution over the whole range of observed fire sizes, particularly at the upper tail. The frequency of large fires often falls far below the expectations under a power law [15,24–27]. For example, in Schoenberg et al. [25], the predicted frequency of very large fires according to a Pareto model fit was orders of magnitude higher than the observed frequency. Yet, reliable prediction of the frequency of these large fires is of fundamental importance given that they generally account for most of the area burned [23], and strongly affect ecosystems dynamics [28]. Schoenberg et al. [25] introduced an alternative distribution, the tapered Pareto, which offers some advantages over a strict power-law or Pareto model. The tapered Pareto distribution closely approximates the Pareto distribution up to some size limit after which the probability density decreases exponentially. This allows a more accurate representation of the upper tailed behaviour of FSDs. Regional variations in the upper tail behaviour of FSDs have also been found [24,29], but we know of no studies that have modelled this variation in relation to environmental factors. Moritz et al. [30] proposed that parametric models, in which the distributional parameters are functions of environmental covariates, could be used to shed light on the effect of these factors on FSDs. We know of no prior studies having done so other than Cumming [15], who showed that land-cover covariates affected FSDs in boreal forest of western Canada. In this study, we extend Cumming’s results to account for fire weather effects, and simultaneously to explore controls on upper tail behaviour fire. We do this by adopting a tapered Pareto distribution, where both the shape and the taper the taper parameter are functions of covariates for land-cover and annual fire weather.

We aimed to assess and contrast how the FSD would respond to changes in two primary abiotic and biotic drivers, namely fire weather and land cover. Our objective was to determine the relative importance of fire weather and land-cover on fire sizes overall and on the frequency of large fires in particular, i.e., on the upper tail behaviour of the FSD. We wanted to evaluate whether fire weather and land-cover would affect the lower and middle parts of the FSD or the tail, both or none, and to what extent extremes of weather or specific vegetation types might shift the FSD towards a higher or lower frequency of larger fires. Using the tapered Pareto distribution, we developed parametric statistical models to link the FSD with both of these controls. We conducted separate analyses for the distribution of lightning- and human-caused fires sizes.

## Methods

### Study area

**Fig 1:**
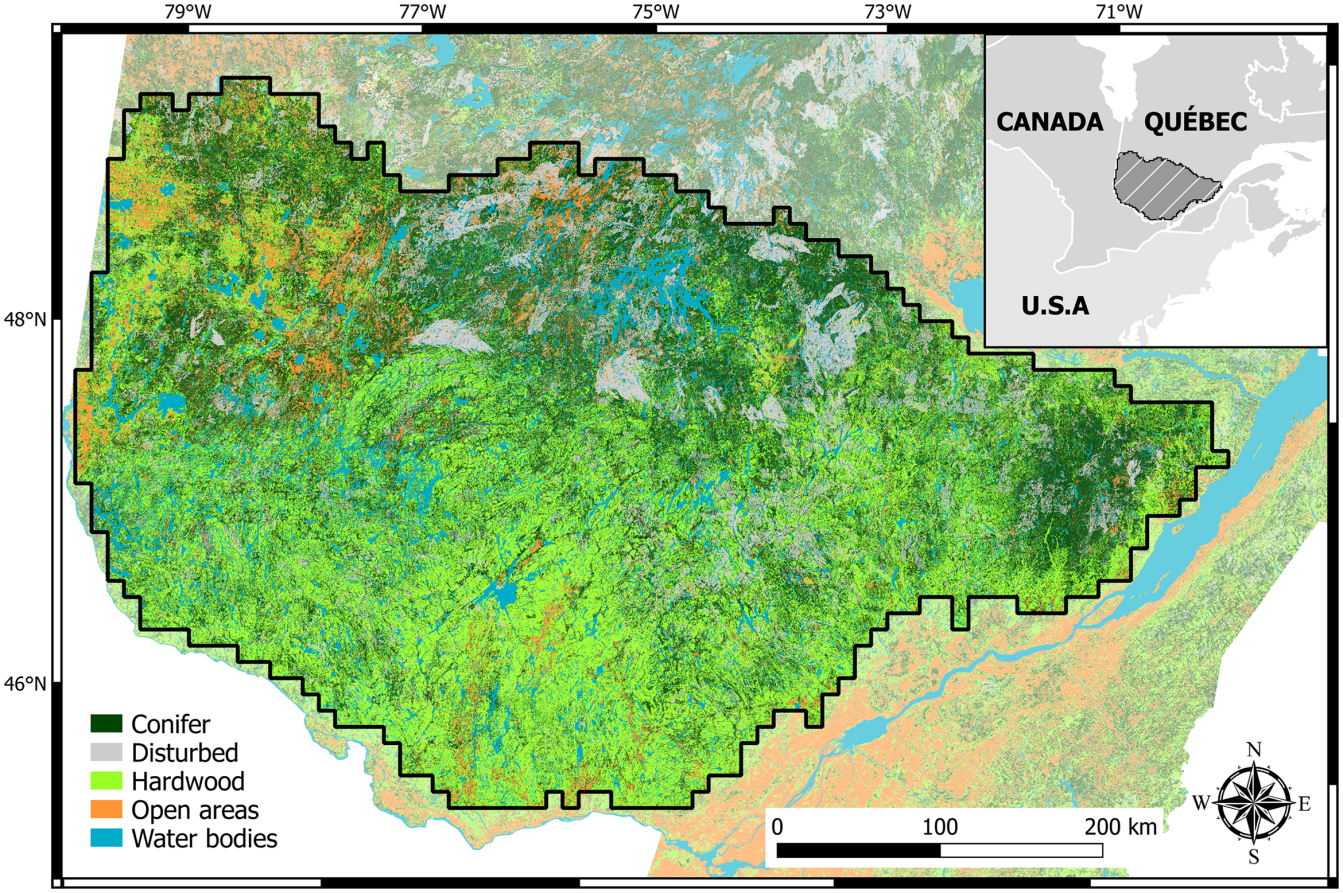
Land-cover map with the five classes used in this study.

The study area (Fig 1) is a 197,000 km^2^ heavily forested region of southern Quebec, Canada. Forests covered about 73% of the area, followed by lakes and large rivers (~10%), open areas (wetlands, croplands and zones with human development; 9%), while recently disturbed areas (≤ 15 years) accounted for the remaining 8%. The topography is gently rolling with elevations of 250–450 m. The climate is humid continental [31]. Climate and land-cover form the two main environmental gradients in our study area. Regional mean temperatures range from –15°C in winter (December, January, February) to +18°C in summer (June, July, August). Seasonal rainfall decreases from east to west and averaged 88–407 mm in winter and 153–547 mm in summer. Sugar maple (*Acer saccharum* Marsh.) and yellow birch (*Betula alleghaniensis* Britt.) are the dominant species in the southern sections. Northwards, forests become gradually dominated by boreal species such as black spruce (*Picea mariana* (Mill.) B.S.P.), balsam fir (*Abies balsamea* (L.) Mill), jack pine (*Pinus banksiana* Lamb), paper birch (*Betula papyrifera* Marsh.) and trembling aspen (*Populus tremuloides*). Forest management is the dominant land use in most of the study area, with limited conversion to agricultural activities on the southern perimeter and a few other areas with appropriate soils.

### Data

We compiled data on fire occurrence and size (source: Ministère des Forêts, de la Faune et des Parcs), fire weather and land-cover for the period 2000–2010. The details of the dataset construction are given in Marchal et al. [10]. Fire attributes include the date of detection, starting location, cause and final size. Fire sizes were measured by SOPFEU, the fire protection agency of Québec. They do this from post-fire aerial photography to delineate the burned area. The data were registered by starting location to a grid of 1969, 10 km square pixels, as used for downscaling climate projections [32]. The study area was entirely embedded in a zone of intensive fire management. We assumed a constant fire detection efficiency over the study spatial and temporal frames given the high capabilities of the fire protection agency [33]. We used Monthly Drought Code (MDC) as an indicator of fire weather conditions because it was found to be well correlated with annual area burned [34]. MDC is the monthly mean of a daily Drought Code, an indicator of the net effect of cumulative daily precipitation and temperature on the moisture content of coarse fuels and organic soils horizons. We estimated MDC for May, June, July and August of each cell in each year, using methods of Bergeron et al. [35]. We also derived seasonal variants of MDC by computing mean MDC for 2, 3 and 4 month periods within years. We used a 5-way land-cover classification (hardwood, conifer, recently disturbed, open areas, open water) derived from digital 1:20 000 vegetation maps following Marchal et al. [10]. The underlying spatial resolution of the land-cover polygons depends on the mapping standard, which vary from 0.1 to 8 ha [36]. We calculated the proportional class areas for each cell in each year. In brief, the classification used forest stand origin dates within the 15 preceding years to determine recently disturbed areas, and a 50% threshold in tree basal area to determine the hardwood and conifer types. For each 10 km square pixel, the proportions covered by all cover types sum to 1.

### Tapered Pareto distribution

The survival function of the tapered Pareto distribution is given by

$$\text{S}\left(x\right)\text{=}{\left(\frac{a}{x}\right)}^{\text{β}}\text{exp}\left(\frac{a-x}{\theta }\right)\text{, }\;a\;\leq x<\infty$$ (1)

where *α* the lower truncation point is generally known a priori, the shape parameter, *β*, controls the rate of frequency decrease as *x* increases and the taper parameter, *θ*, governs the location of the exponential taper. The survival function of the tapered Pareto begins to decay exponentially near a fire size of *θ*; the distribution converges to a Pareto distribution as *θ* approaches infinity. An increase in *β* will lead to a more negative (or steeper) slope, therefore smaller fire sizes. An increase in *θ* will lead to a weaker taper, and thus larger fires. The mean (calculated as in [37]) does not uniquely determine the two parameters. Thus, it is possible that two distributions could have the same mean but very different tail behaviours. On a log-log plot, the survival function of a tapered Pareto is approximately linear for a range from *α* to near theta, and then gradually decays in an exponential fashion. We find graphical methods helpful in understanding the influence of the shape and taper parameters on the size distribution (S1 Fig). We fixed *α* = 1 because of the influence of fire suppression on small fires in managed forests [38] such as the study area, and controls distinct from those affecting fire spread determine fire size at this scale [39]. We estimated *β* and *θ* by maximum likelihood.

### Regression analysis

We built a set of alternative hypotheses, here formulated as statistical models where we expressed two parameters of the tapered Pareto distribution, *β* and *θ*, as a function of either fire weather or land-cover, both or neither. The null hypothesis corresponds to the case where neither *β* nor *θ* are influenced by fire weather or land cover. This led us to design a full factorial experiment (see Table 1 for levels). Since not necessarily the same fire weather variables (e.g. different months) would be best correlated with both *β* and *θ*, e.g. because they are not abstractions of the same underlying processes, we introduced some flexibility in that fire weather variables used for *β* and *θ* may differ. However, in order to evaluate our hypotheses on the same basis, models should be nested. In other words, we had to retain a single fire weather variable for all *β*s expressed a function of fire weather, and possibly another for all *θ*s. From there, we selected the fire weather variables present in the best model, itself selected according to the Akaike Information Criterion corrected for small samples (AIC_c_, [40]). We checked for linear and nonlinear correlations among the fire weather and land-cover variables by calculating the Spearman’s rho coefficients.

**Table 1:** Alternate statistical models of fire size distribution ordered by AIC_c_.

Model form	Terms^1^		AIC_c_^2^	AD^3^
	β	θ		
Lightning				
**WLC_WLC**	**WLC**	**WLC**	**1215**	**96.63**
WLC_LC	WLC	LC	1216	98.63
LC_WLC	LC	WLC	1234	103.89
LC_LC	LC	LC	1237	103.25
WLC_W	WLC	W	1256	98.19
WLC_Null	WLC	Null	1258	97.80
W_WLC	W	WLC	1261	139.41
W_LC	W	LC	1262	140.80
LC_W	LC	W	1270	103.85
LC_Null	LC	Null	1270	103.35
Null_WLC	Null	WLC	1277	147.13
Null_LC	Null	LC	1279	144.79
W_W	W	W	1303	143.15
W_Null	W	Null	1303	142.71
Null_Null	Null	Null	1318	152.27
Null_W	Null	W	1319	151.97
Human				
**WLC_WLC**	**WLC**	**WLC**	**1820**	218.35
LC_LC	LC	LC	1822	216.26
WLC_LC	WLC	LC	1828	216.90
LC_WLC	LC	WLC	1829	**215.41**
Null_WLC	Null	WLC	1871	279.97
Null_LC	Null	LC	1872	278.80
W_WLC	W	WLC	1872	274.35
W_LC	W	LC	1873	271.33
LC_Null	LC	Null	1873	218.03
WLC_Null	WLC	Null	1875	218.08
LC_W	LC	W	1875	218.36
WLC_W	WLC	W	1876	218.40
W_Null	W	Null	1909	275.16
Null_Null	Null	Null	1910	∞
W_W	W	W	1911	275.39
Null_W	Null	W	1912	279.02

The best fit by each criteria is shown in bold.

^1^The presence of a fire weather term is noted “W”, land-cover terms “LC” and the intercept only “Null”.

^2^AIC_c_ is the small sample corrected Akaike Information Criterion.

^3^AD is the Anderson Darling statistic modified for upper tail sensitivity (see Methods). For both AIC_c_ and AD, lower values indicate a better fit.

We used a log-linear model for *β* to enforce non-negativity, and because there is a linear relationship between *β* and the logarithm of the fire sizes (see Eq 1). *θ* is on the same scale as the fires sizes, thus we modeled *θ* as a linear function of the covariates. This leads to the following system of equations:

$$\text{log}\left(\beta_{i,t}\right)=\gamma_{0}+\sum_{k=1}^{n-1}\gamma_{k}LC_{k,i,t}+\gamma_{w}W_{i,t}$$ (2)

$$\theta_{i,t}=\delta_{0}+\sum_{k=1}^{n-1}\delta_{k}V_{k,i,t}+\delta_{w}W_{i,t}$$ (3)

where *W*_*i*,*t*_ is the fire weather covariate in pixel *i* in year *t*; *LC*_*k*,*i*,*t*_ is the proportional area of cover type *k* in pixel *i* in year *t*; *γ* and *δ* are vectors of parameters to be estimated; n = 5 is the number of cover types in the model and index *w* is equal to n. These equations are the most general model. The alternate hypotheses are expressed by setting certain coefficients to 0. To ensure identifiability, we dropped the proportions of conifer-dominated stands, thus *k* ends at *n* − 1. The intercepts can be interpreted as the conifer-dominated reference level against which coefficients for the other land-cover terms can be compared. We measured the relative importance of environmental covariates on *β* and *θ*, and by extension on the FSD, by comparing their effect sizes. To facilitate the comparisons of effect sizes, we normalized the fire weather variables by scaling them between 0 and 1, so that *x* = (*x*–*x*_*min*_) / (*x*_*max*_−*x*_*min*_). This ensures that all covariates are on the same scale; land-cover terms being proportions were already bounded between 0 and 1. We rescaled the fire weather variables independently for the models of lightning- and human-caused fires.

We estimated *β* and *θ* by direct minimization of the log-likelihood function (Eq 4; taken from [40]) using the differential evolution algorithm implemented in the DEoptim package [41] for the R software [42]:

$$\text{log}L\left(\beta_{i,t},\theta_{i,t}\right)=\sum_{j=1}^{N}\text{log}\left(\frac{\beta_{i,t}}{x_{j}}+\frac{1}{\theta_{i,t}}\right)+\beta_{i,t}N\;\text{log }a-\beta_{i,t}\sum_{j=1}^{N}\text{log}x_{k}+\frac{aN}{\theta_{i,t}}-\frac{1}{\theta_{i,t}}\sum_{j=1}^{N}x_{k}$$ (4)

where *N* is the number of fires, and *i*, *t* are as in Eq 2. We assessed models goodness of fit using AIC_c_ score and the Anderson-Darling statistic (AD) modified for upper tail sensitivity [43]. For the best models of lightning- and human-caused fires, we used a parametric bootstrap approach to derive 95% confidence intervals around the estimates. We generated 5,000 replicate datasets by random sampling from the tapered Pareto distributions predicted using the parameter estimates from the best models. For each replicate, we re-estimated the model parameters, and took the 2.5 and 97.5% percentiles of the bootstrapped estimates as our CIs. We computed the confidence bounds around the empirical survival function as in Schoenberg & Patel [44]. We used Eq 9 from Kagan & Schoenberg [37] to calculate expected fire sizes for each cell and year.

## Results

Over the 11-year study interval, 186 lightning-caused fires and 397 human-caused fires were recorded. Fire sizes ranged from 1 to 107,004 ha for lightning fires, and from 1 to 59,847 ha for human-caused fires. The mean and median sizes were 994 ha and 3 ha for lightning-caused fires, and 208 ha and 2.3 ha for human-caused fires. There were no strong correlations between land-cover and monthly fire weather at the spatial and temporal scales of the analysis (S1 Table).

**Fig 2:**
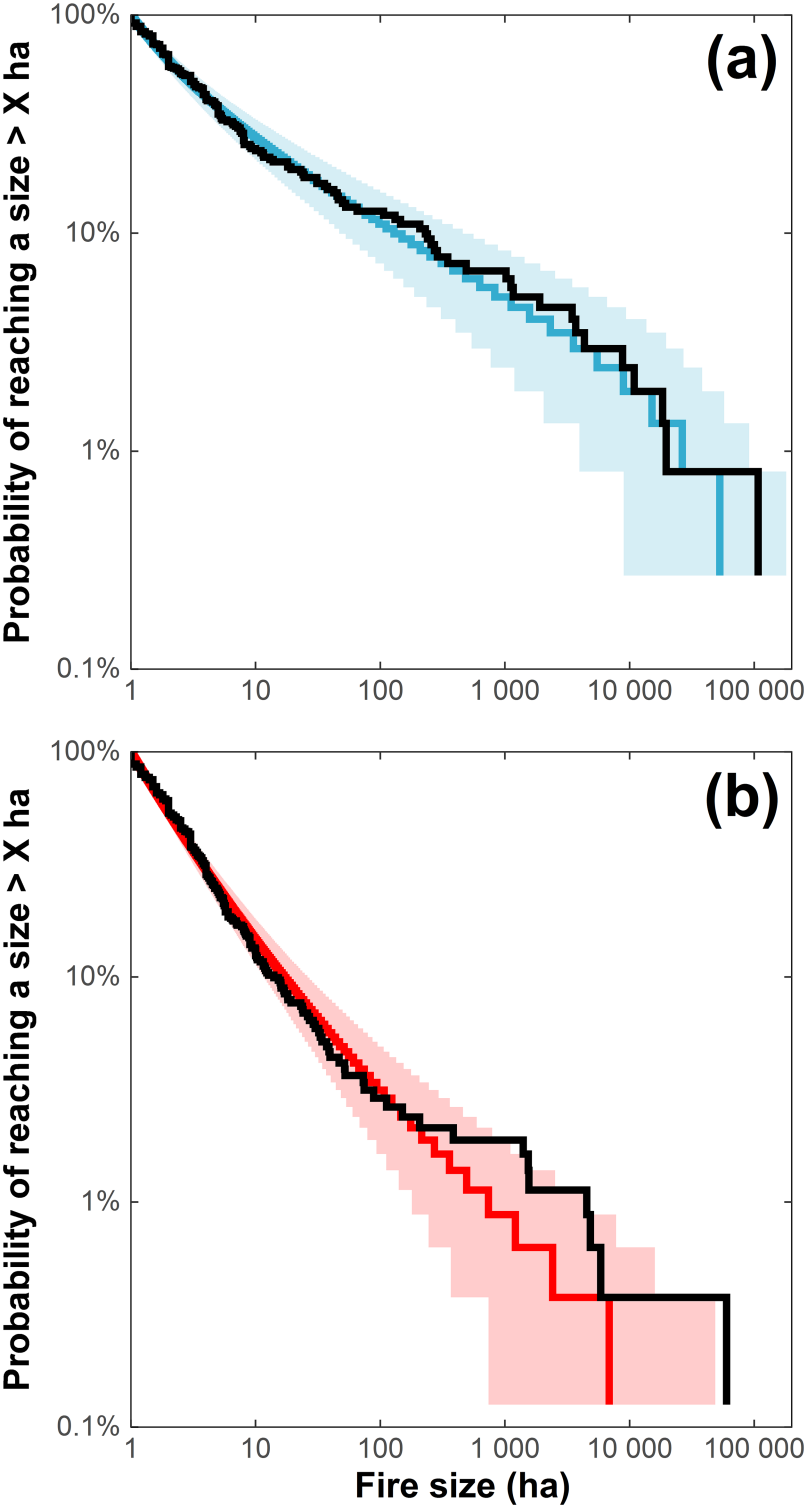
Fitted survival functions of (a) lightning- and (b) human-caused fire sizes) with 95% confidence intervals (shaded polygons), and empirical distributions (black lines).

For lightning-caused fires, the best model included both fire weather and land-cover on both *β* and *θ* (Table 1). Models with fire weather terms alone did not perform as well as models with land-cover terms alone, and in some cases did not perform any better than the null model. Further, dropping the fire weather term did not lead to a huge drop in model support. For example, removing the fire weather term on both sides (LC_LC model) led to an AIC_c_ increase of 22 while removing the land-cover terms instead (W_W model) increased AIC_c_ by 88. Our best models described FSDs reasonably well, with the tail tapering as do the data but also capturing to some extent the upward curvature present in the first part, i.e. left portion, of the FSD (range 10 to 1,000 ha; Fig 2).

For human-caused fires, the best model included both fire weather and land-cover on both *β* and *θ* (Table 1). The addition of fire weather covariates to linear predictors did not necessarily lead to better support compared to models with land-cover terms only: the model having fire weather and land-cover (WLC) on both *β* and *θ* sides was equally supported as that having land-cover alone (LC) (ΔAIC_c_ ≤ 2, Table 1). Dropping the land-cover terms on both *β* and *θ* sides led to an AIC_c_ increase of 91 while dropping fire weather led to an AIC_c_ increase of only 2. All observations made with AIC_c_ were partially or totally corroborated by the AD statistic (Table 1).

**Fig 3:**
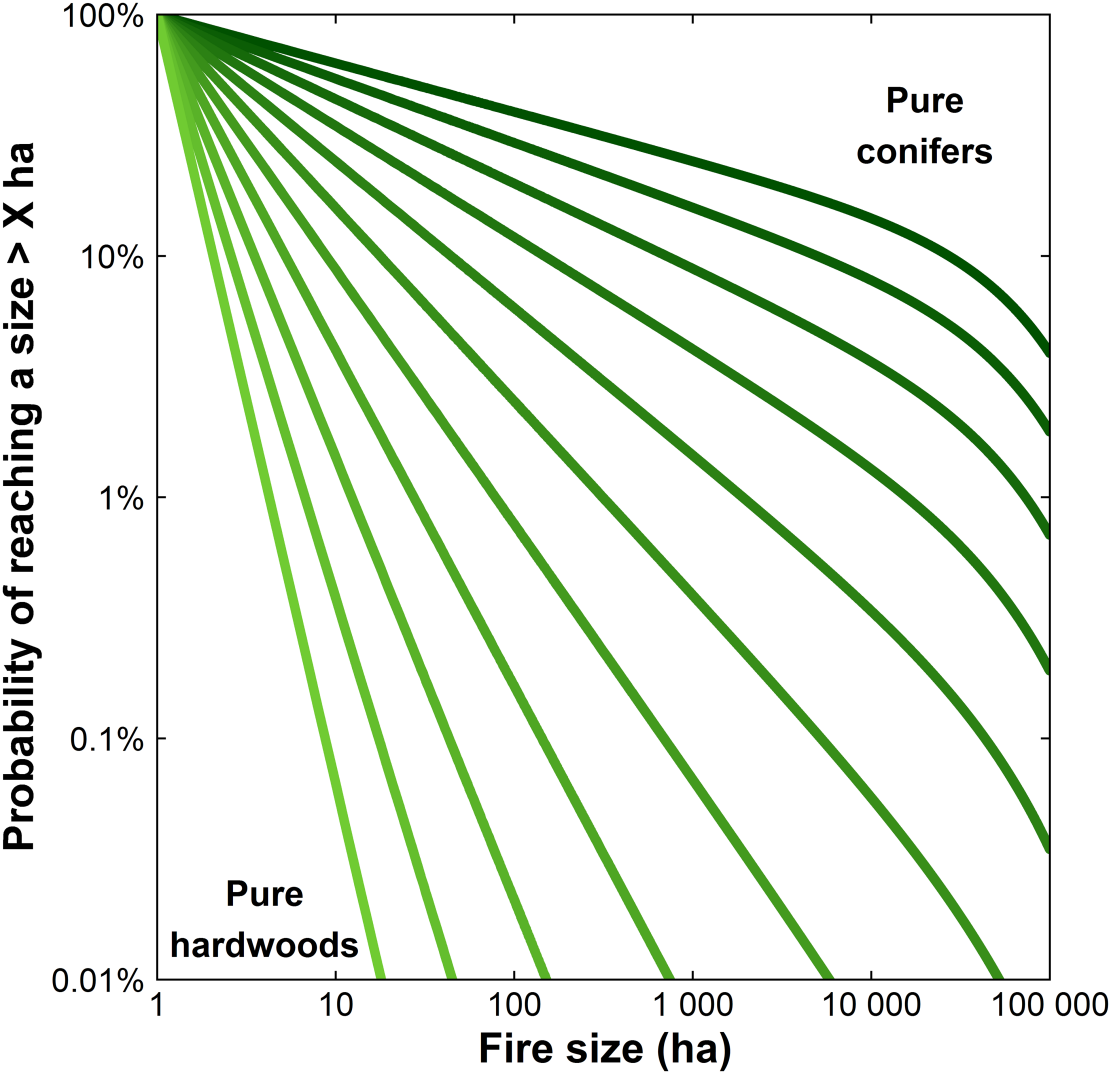
Influence of landscape fuel composition on the shape of the predicted distribution of lightning-caused fires sizes under the best supported model (full model where β and θ are both function of fire weather and land cover, see Tables 1 and 2). Predicted FSDs for hypothetical landscapes from 100% conifer to 100% deciduous in 10% increments, under means of fire weather covariates.

Both lightning- and human-caused FSDs responded to an increase in the proportion of hardwood-dominated stands or water bodies in the landscape by increasing0020*β* and decreasing *θ* (Table 2), thus squeezing FSDs towards a smaller range of sizes (Fig 3). The abundance of hardwood-dominated had the strongest “landscape effect” on the shape parameter *β*, which determines the decay of frequency with size. Hardwood-dominated stands influenced FSDs in essentially the same degree as water bodies (*γ*_*HW*_ = 2.76 and *γ*_*WT*_ = 2.58 for lightning-caused FSD, and *γ*_*HW*_ = 1.66 and *γ*_*WT*_ = 1.57 for human-caused FSD, Table 2). The influences of hardwood-dominated stands and water bodies on the slopes of FSDs, i.e. *β*s, were statistically distinct from the other vegetation types in that their coefficients did not overlap with that for conifer-dominated stands (95% CI, Table 2), and likely distinguishable from 0 given their effect sizes and the lower bound of their CIs. Thus, we reject the null hypothesis that *β* is independent of land cover classes.

**Table 2:** Maximum-likelihood estimates and 95% confidence intervals for each term in top models for lightning- and human-caused fires: models WLC_WLC and WLC_WLC of Table 1.

Term	Estimate	Confidence interval (95%)
Lightning		
β		
*γ*_0_	-1.02	(-1.74, -0.40)
*γ*_*MDC*_*Jun*_	-1.51	(-2.23, -0.74)
*γ*_*HW*_	2.76	(1.88, 3.67)
*γ*_*D*_	1.05	(-0.71, 2.83)
*γ*_*O*_	1.18	(-1.25, 4.16)
*γ*_*WT*_	2.58	(1.07, 5.02)
θ (x10^5^)		
*δ*_0_	1.5	(0.11, 4.12)
*δ*_*MDC*_*MayJun*_	-1	(-2.37, 0.45)
*δ*_*HW*_	-0.16	(-1.91, 1.79)
*δ*_*D*_	-1.36	(-5.36, 0.4)
*δ*_*O*_	-0.99	(-3.07, 1.76)
*δ*_*WT*_	-0.74	(-2.50, 0.00)
Human		
β		
*γ*_0_	-1.09	(-1.83, -0.45)
*γ*_*MDC*_*MayJul*_	-0.098	(-0.59, 0.39)
*γ*_*HW*_	1.66	(0.90, 2.55)
*γ*_*D*_	-0.78	(-2.40, 1.50)
*γ*_*O*_	0.78	(-3.36, 2.14)
*γ*_*WT*_	1.57	(0.30, 3.03)
θ (x10^4^)		
*δ*_0_	-0.67	(-3.47, 0.76)
*δ*_*MDC*_*May*_	1.59	(-2.63, 5.71)
*δ*_*HW*_	2.9	(0.05, 16.4)
*δ*_*D*_	-2.91	(-15.34, -0.02)
*δ*_*O*_	17.7	(0.59, 82.4)
*δ*_*WT*_	0.8	(-0.73, 5.46)

Coefficients with CI that encompasses zero are not statistically significant. Estimates with 0 subscripts indicates intercepts and HW, hardwood; D, recently disturbed; O, open areas; WT, open water.

**Fig 4:**
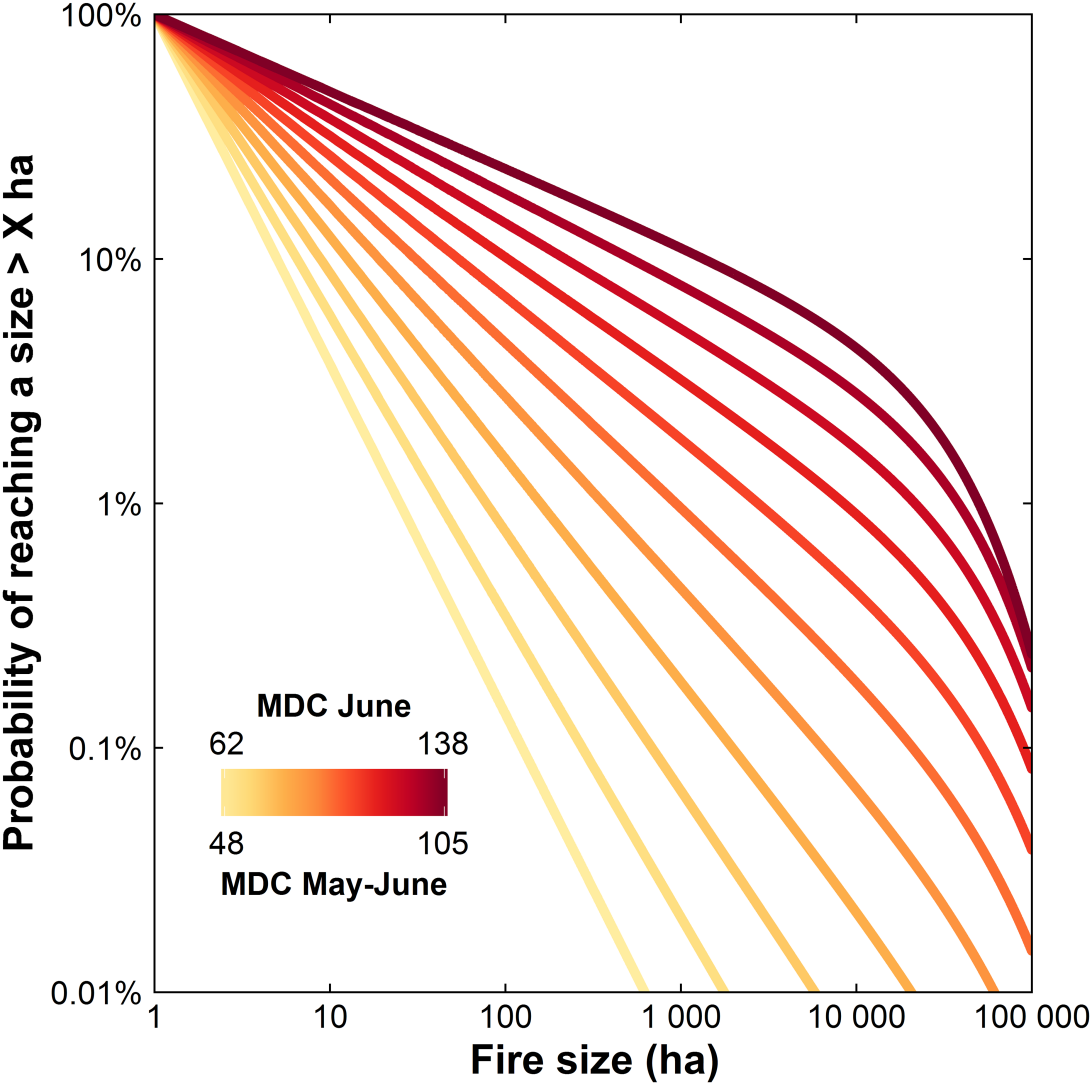
Influence of fire weather on the shape of the expected distribution of the lightning-caused fires sizes (full model where β and θ are both function of fire weather and land cover, see Tables 1 and 2). Predicted FSDs for fire weather conditions in 10 percentiles increments from the driest to wettest Monthly Drought Code (MDC) recorded in our data. For illustrative purposes, all lines are for landscapes with 50% hardwoods and 50% conifer-dominated stands.

For the lightning-caused FSD, both *β* and *θ* decrease with more extreme fire weather (*γ*_*MDC*_*Jun*_ = −1.51, *δ*_*MDC*_*MayJun*_ = −1, Table 2). Decreasing *β* implies an increasing probability of large fires below the taper-size *θ*, while decreasing *θ* sharply decreases the probability of fires larger than *θ* (Fig 4). For the human-caused FSD, under increasingly extreme fire weather, *β* tends to decrease slightly (*γ*_*MDC*_*MayJul*_ = −0.098), while *θ* shifts towards larger fire sizes (*δ*_*MDC*_*May*_ = 1.59, Table 2).

**Fig 5:**
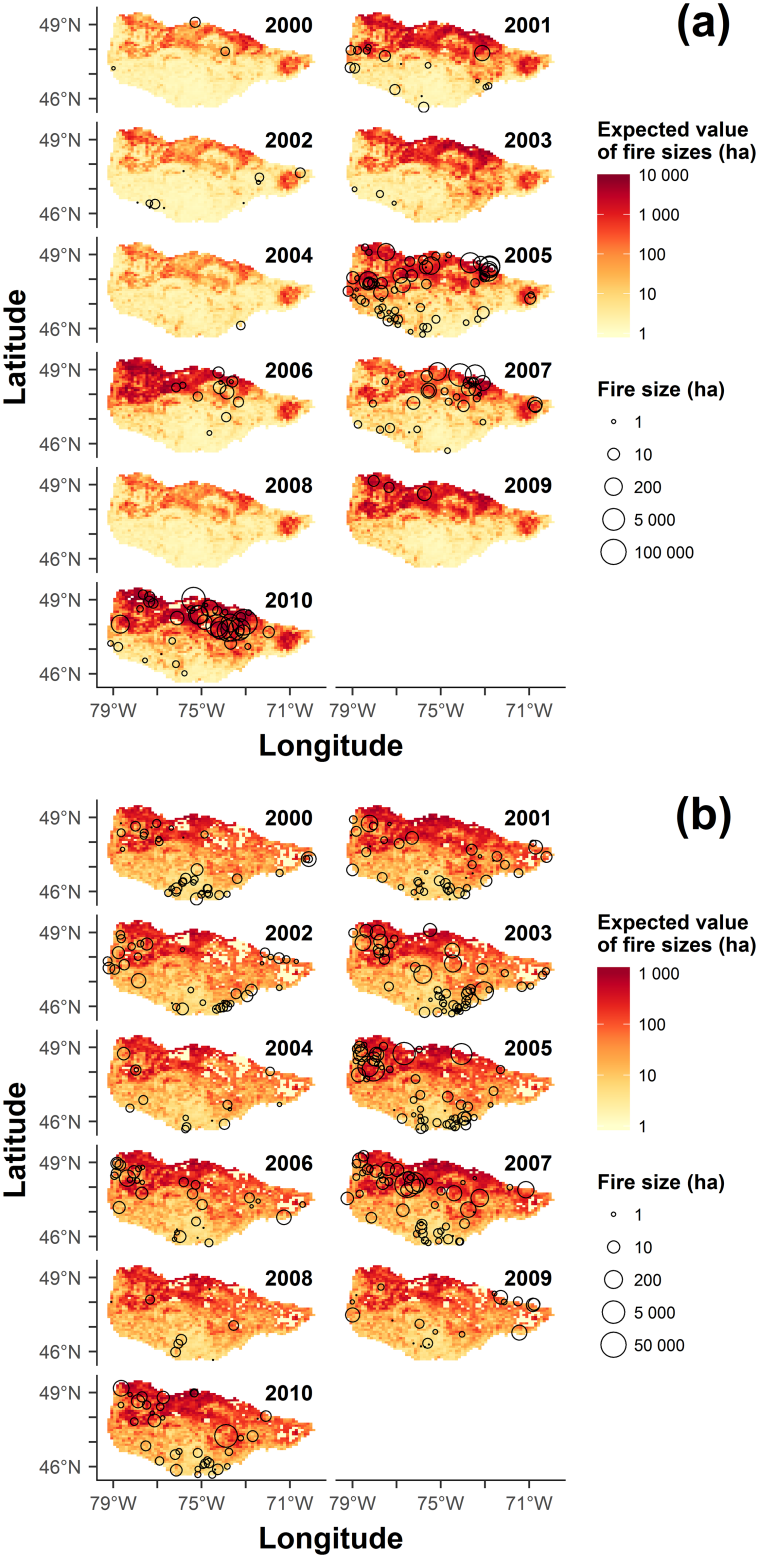
Expected fire sizes, conditional on the fitted tapered Pareto distributions of (a) lightning- and (b) human-caused fires along with the location and sizes of recorded fires (black circles).

The latitudinal gradient in land-cover composition, with conifer abundance increasing with latitude (S2 Fig), is clearly reflected in the spatial patterning of the predicted mean fire size (Fig 5). The annual variability in the MDC of June is presented in S3 Fig. To explore the implications of our results with respect to the potential effects of fuels management, we compared the predicted frequencies of large fires on landscapes of 100% conifer-dominated stands and “treated” landscapes with 70% conifer and 30% hardwood. According to the best-supported model (WLC_WLC), under mean fire weather conditions the probability of a lightning-caused fire exceeding 100,000 ha on the treated landscape is reduced by a factor of 21, relative to the untreated landscape. In extreme fire weather conditions, i.e. the 90^th^ percentiles of fire weather covariates, this effect size is reduced to a factor of 7 (S4 Fig). In landscapes fully covered by hardwoods and under mean fire weather conditions, fires larger than a few tens of hectares are predicted to be very improbable (Fig 3).

## Discussion

Fire weather has been considered the dominant control on the FSD in boreal forests, but recent studies have shown that fire weather alone is not enough to determine either fire spread [6], nor the final fire sizes [13,19,20]. Other factors such as human footprint and topography ([6], Table 3) or land-cover [13] are also important. We found that land-cover was more important than monthly fire weather as a control of FSDs in Southern Québec (*β*s, Table 1; Figs 3 and 4). While many coefficients of the land-cover terms are not distinguishable from 0 at the 95% level (*γ*s, *δ*s, and their CIs, Table 2), models with land-cover terms only were much more strongly supported based on both AIC_c_ and AD statistics than were models with fire weather terms only (Table 1). Our results support the hypothesis that fires preferentially burn some fuel types and avoid others, and in this respect are consistent with past findings [18–20], excepting of Podur and Martell [45]. Because past disturbances reduce the availability of fuel to burn, subsequently limiting the ability of future wildfires from spreading [14,15], neglecting land-cover effects, including negative feedbacks where they exist, may result in unreliable fire risk forecasts.

According to Liu *et al*. [20] and Barros & Pereira [19], the dominant control of the FSD shifts from land-cover to fire weather with increasing fire sizes. If so, one could expect that models where the taper parameter *θ* was a function of fire weather covariates would be better supported than models where it was a function of land-cover covariates. We found the contrary (Table 1). Further, the effect sizes of the land-cover terms were typically higher than those of fire weather terms. Most of the effect sizes of the fire weather terms did not differ significantly from 0 (Table 2), which suggests a limited effect of monthly fire weather. Particularities of our study region and 11-years dataset may partially explain these results. Under sufficiently extreme fire weather conditions, all fuel types can support high intensity fire with rates of spread such that we would expect little effect of land-cover on fire size. However, in the study region drought conditions are rarely severe (see Fig 4 in Girardin and Wotton [34] for Canada-wide variations of MDC), and extreme MDC values are infrequent. In real landscapes there are probably interactions between land-cover and fire weather. Fuels types differ in terms of fuel loads and drying rates [46] such that they exhibit different fire behaviours except perhaps under the most extreme conditions.

For lightning-caused FSD, the taper location shifted towards lower sizes under more extreme fire weather. While this may seem counterintuitive, our graphical analysis (Fig 4) revealed that under low MDC values, the tapered Pareto is converging to a Pareto distribution with a large slope (β high) and low expected size. Large fires are so improbable under these conditions that there is no evidence of tapering in the distribution under these conditions. Tapering, reflecting larger scale controls on fire size such as geography or burning season length, becomes important only under drier conditions (Fig 4).

Our study used 11 years of data, which is a limited time frame. While our sample size of fires was reasonable (186 caused by lightning and 397 by humans) compared to previous studies (e.g. [18,20,45]), the lack of data at the extreme upper tail of the FSD should encourage continued modelling efforts. Although there was considerable variation in conditions across the years, our ability to infer the relationship between the FSD, land-cover and fire weather would be enhanced with more years of data and further our ability to predict reliably in conditions not observed. For example, in the case of the human-caused fires, our best models did not correctly capture the tail. However, this could also be the signature of processes or environmental controls not included in this study, e.g. the growing season start date because more than 70% of human-caused fires started in April or May (S5 Fig). At that time, hardwood foliage is absent and hardwoods are much more flammable than during the summer season [47, 48]. The MDC covariate may to account for these conditions. Our results use monthly drought code to represent fire weather; therefore our conclusions do not reflect the short-term (hourly or daily) variations in fuel flammability, and may underestimate the influence of fire weather. This study provides inference only about longer term and larger scale characteristics of fire regime rather than individual fires. Finally, we assumed a particular error distribution (tapered Pareto) and functional form for the model parameters in relation to the covariates. The choice of error distribution is well supported empirically (e.g. [25]). We feel our choice of functional forms (linear in the shape parameter, log-linear in the taper parameter) are justified, but it remains possible that the model structure is misspecified. Alternate modelling approaches that allowed for more nonlinear relationships or for interactions might yield different results in terms of the relative importance of different classes of covariates.

We explicitly integrated the effects of land-cover on fire regimes via the coefficients for fuel-type in the models. This would enable forecast of FSDs based on these models to respond to land-cover feedbacks. This integration is essential (a) in assessing vulnerabilities to fire in a context of climate change [49,50] and subsequent adaptation strategies, (b) for the sustainable long-term management of forest resources [13,51], and (c) to improve our understanding of the climate–fire–fuels linkages and more generally improve the understanding of fire as a key process of the ecosystems on Earth [1]. This study constitutes the first step towards improving the reliability of FSDs’ forecasts, which will contribute directly to decision support systems, coupled climate–fire–fuels modeling and a more mechanistic vision of the linkages between fire, weather and land-cover. We stress the need to account for land-cover while forecasting FSDs, particularly in a context where land-cover changes are expected due to both climate change (e.g. [52]) and steadily increasing levels of land-cover conversion. Our findings have implications for fire risk management in that fuels may be managed to reduce fire risk. For example, converting 30% of a conifer-dominated landscape to hardwoods will, according to our models, substantially decrease the size of fires (Fig 3 and S4 Fig) along with the number of fires [10].

## Supporting information

S1 Fig Influence of (a) *β* and (b) *θ* parameters on the shape of the tapered Pareto survival function. For illustrative purposes, we fixed *θ* at 100,000 in (a) and *β* at 0.5 in (b).
(PDF)

S2 Fig Spatial variation in the proportional abundance of the conifer-dominated forest (%). (PDF)

S3 Fig Annual variations of the Monthly Drought Code (MDC) of June for the period 2000–2010. (PDF)

S4 Fig Influence of landscape fuel composition on the shape of the predicted distribution of lightning-caused fires sizes under the best supported model (full model where β and θ are both function of fire weather and land cover, see Tables 1 and 2). Predicted FSDs for hypothetical landscapes from 100% conifer to 100% deciduous in 10% increments, under extreme fire weather: 90^th^ percentiles of fire weather covariates.
(PDF)

S5 Fig Histogram of the starting date (month) of lightning- (blue bars) and human- (red bars) caused fires during the 2000–2010 period. (PDF)

S6 Fig Estimated survival functions for all competitive hypotheses tested (black lines) with their 95% confidence intervals (grey lines) along with empirical survival function for the distribution of lightning-caused fires sizes (blue lines). *β*_0_ and *γ*_0_, intercepts; W, fire weather and V, land-cover terms.
(PDF)

S7 Fig Estimated survival functions for all competitive hypotheses tested (black lines) with their 95% confidence intervals (grey lines) along with empirical survival function for the distribution of human-caused fires sizes (red lines). *β*_0_ and *γ*_0_, intercepts; W, fire weather and V, land-cover terms.
(PDF)

S8 Fig Influence of landscape fuel composition on the shape of the predicted distribution of lightning-caused fires sizes under the best supported model (full model where β and θ are both function of fire weather and land cover, see Tables 1 and 2). Predicted FSDs for hypothetical landscapes from 100% conifer to 100% disturbed in 10% increments, under means of fire weather covariates.
(PDF)

S1 Table Correlation matrix of the Spearman’s rho coefficients. **HW**, hardwood; **CN**, coniferous; **D**, recently disturbed; **O**, open areas; **WT**, open water.
(PDF)

S1 File Raw data. (RDATA)
