# Correction: Land cover, more than monthly fire weather, drives fire-size distribution in Southern Québec forests: Implications for fire risk management

**DOI:** 10.1371/journal.pone.0185515

**Published:** 2017-09-21

**Authors:** Jean Marchal, Steve G. Cumming, Eliot J. B. McIntire

The following information is missing from the Funding section: This study was supported by the Canadian Statistical Sciences Institute.
