# Supplementary figures and images for: Land cover, more than monthly fire weather, drives fire-size distribution in Southern Québec forests: Implications for fire risk management
Source: PLoS One. 2017 Jun 13;12(6):e0179294. doi: 10.1371/journal.pone.0179294 (PMC5469487; doi:10.1371/journal.pone.0179294)

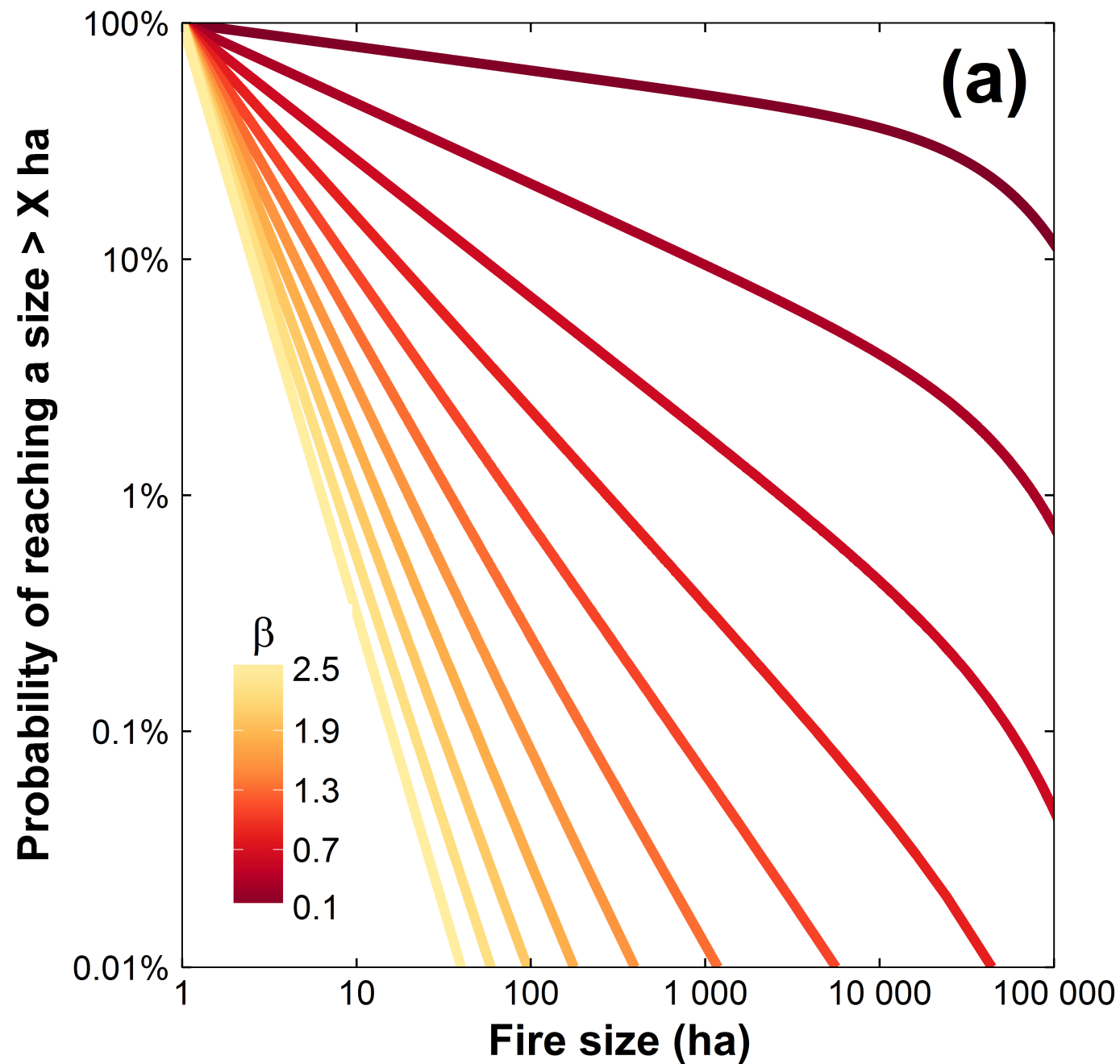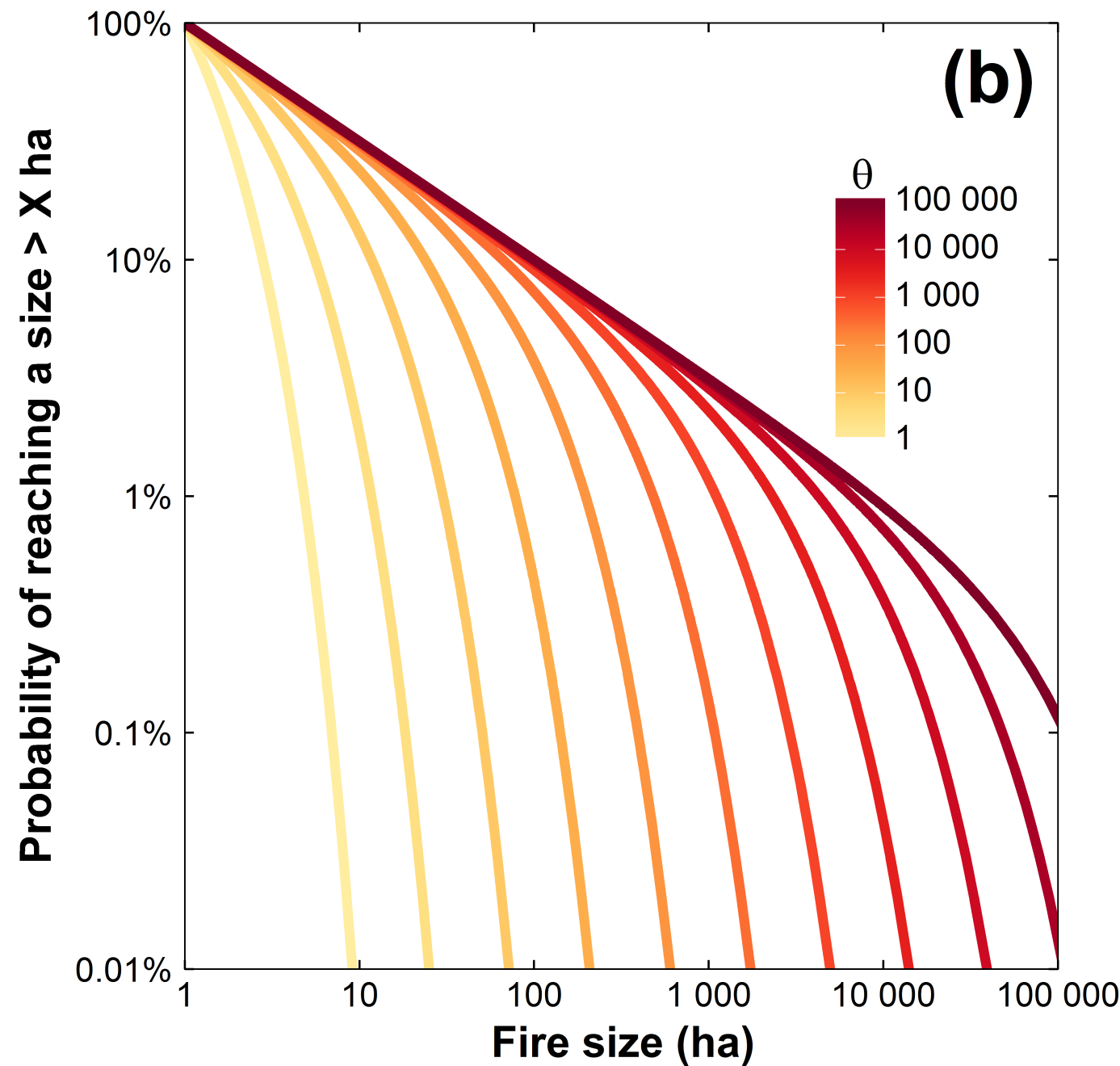

Supplement: S1 Fig — For illustrative purposes, we fixed θ at 100,000 in (a) and β at 0.5 in (b). (PDF) [file pone.0179294.s001.pdf]

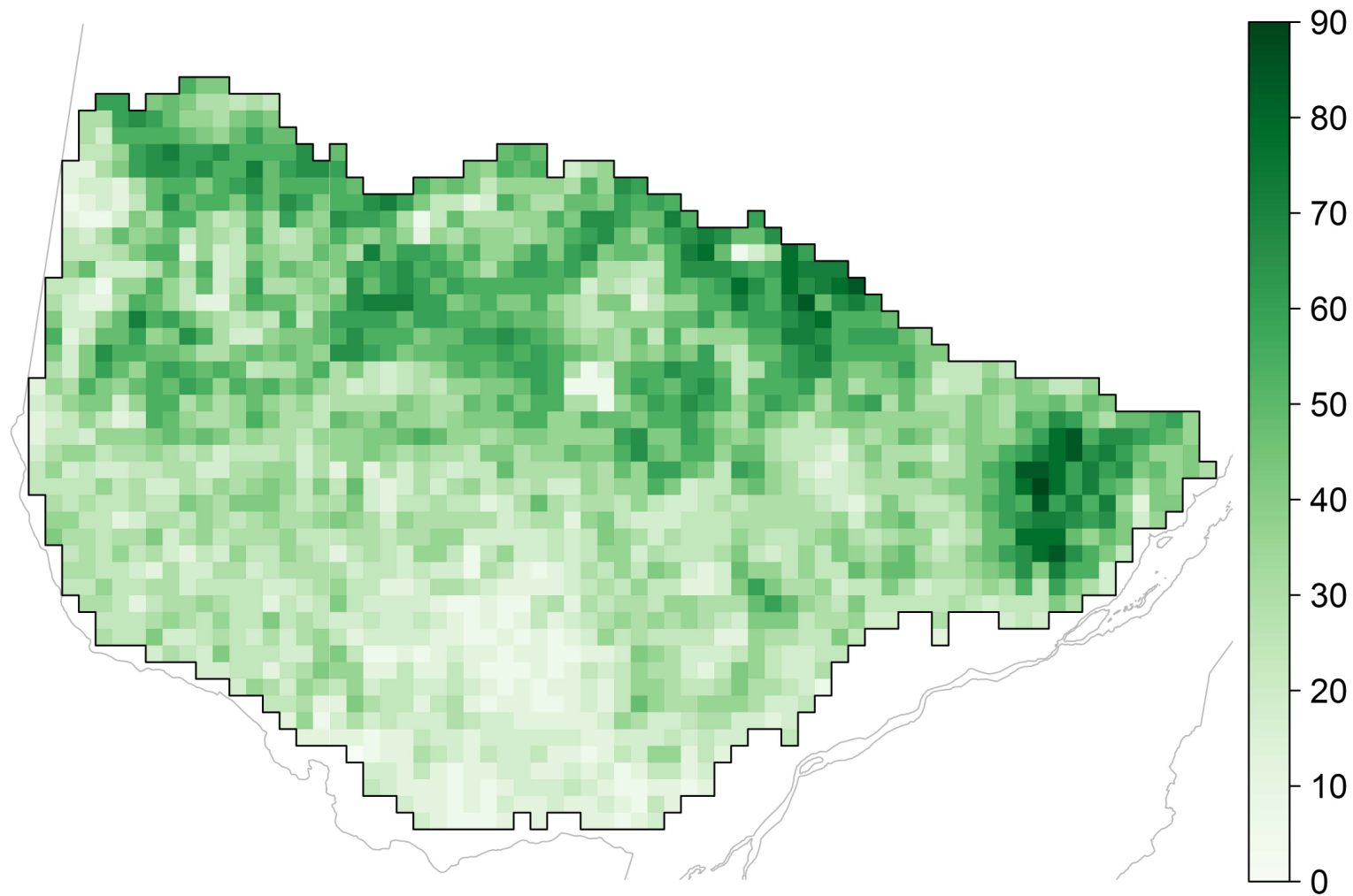

Supplement: S2 Fig — (PDF) [file pone.0179294.s002.pdf]

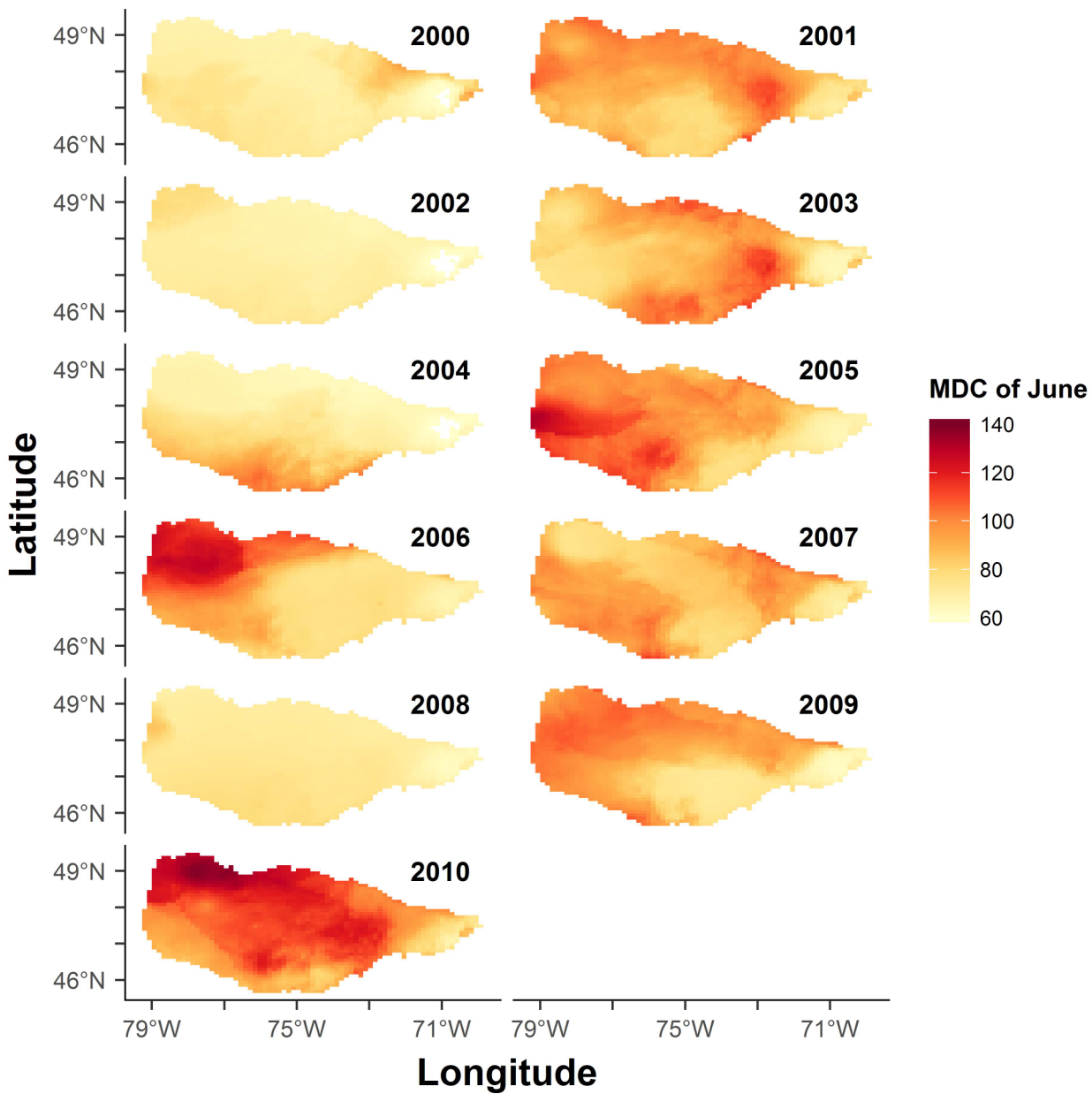

Supplement: S3 Fig — (PDF) [file pone.0179294.s003.pdf]

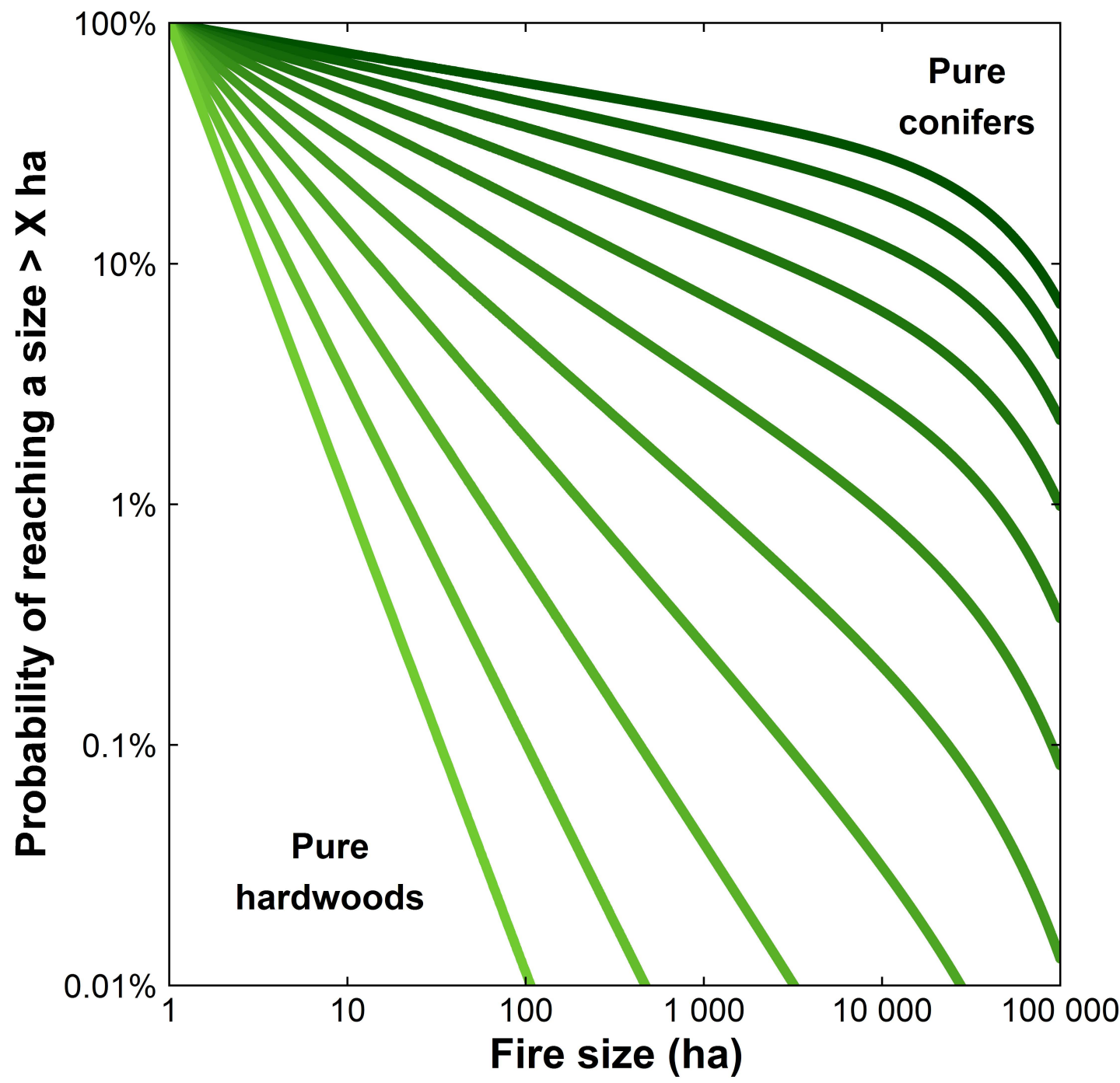

Supplement: S4 Fig — Predicted FSDs for hypothetical landscapes from 100% conifer to 100% deciduous in 10% increments, under extreme fire weather: 90th percentiles of fire weather covariates. (PDF) [file pone.0179294.s004.pdf]

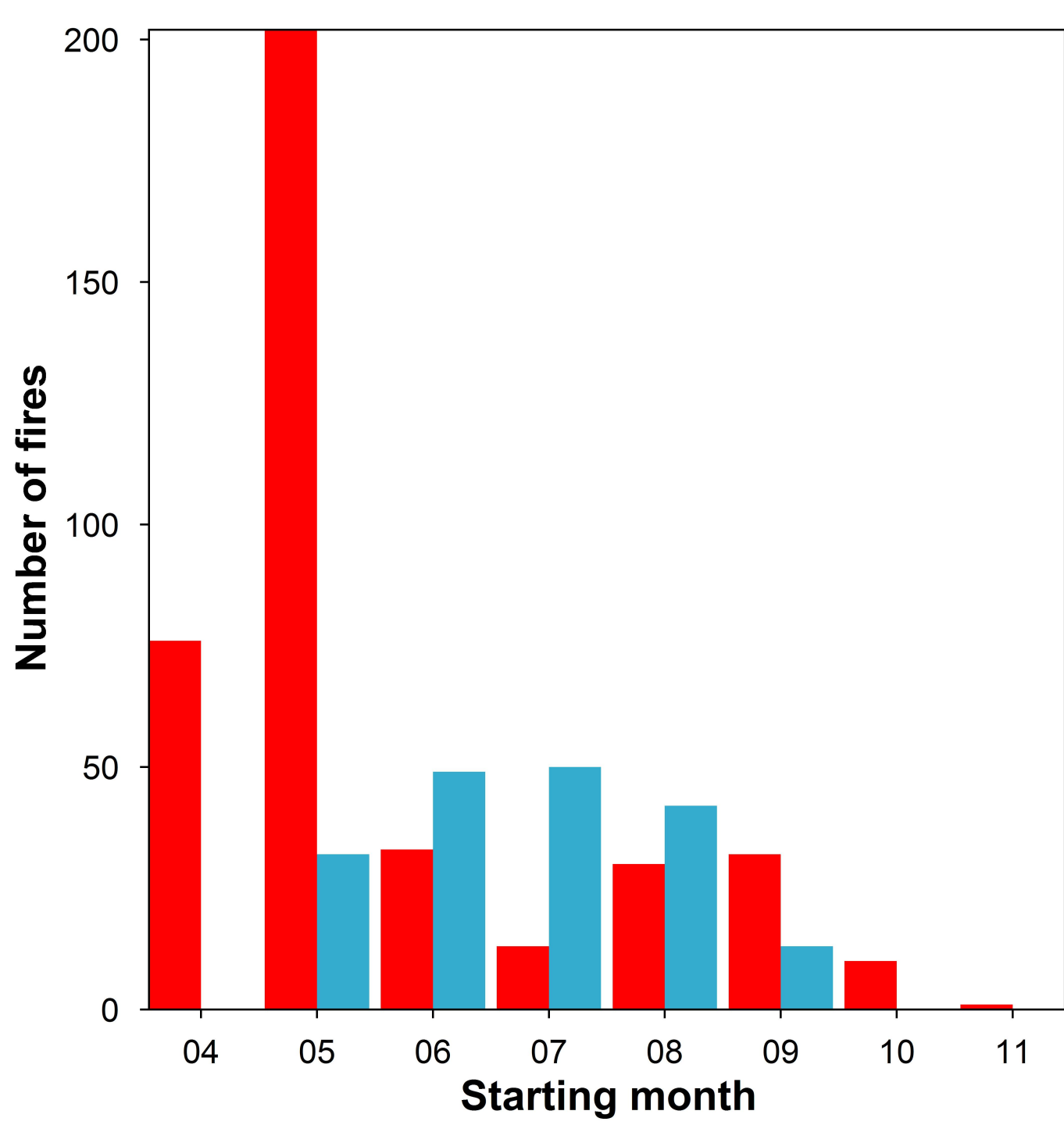

Supplement: S5 Fig — (PDF) [file pone.0179294.s005.pdf]

Probability of reaching a size > X ha

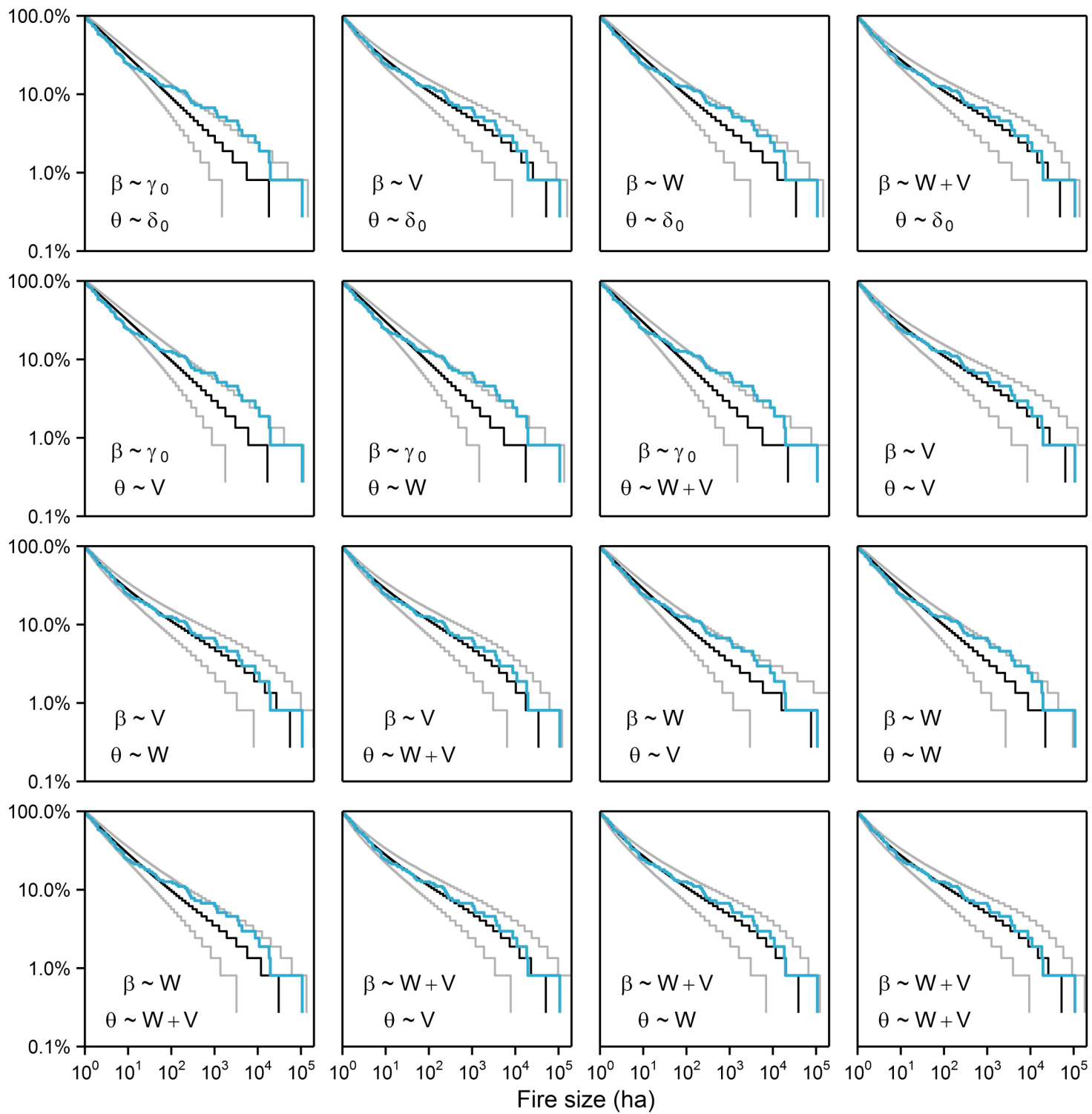

Supplement: S6 Fig — β0 and γ0, intercepts; W, fire weather and V, land-cover terms. (PDF) [file pone.0179294.s006.pdf]

Probability of reaching a size > X ha

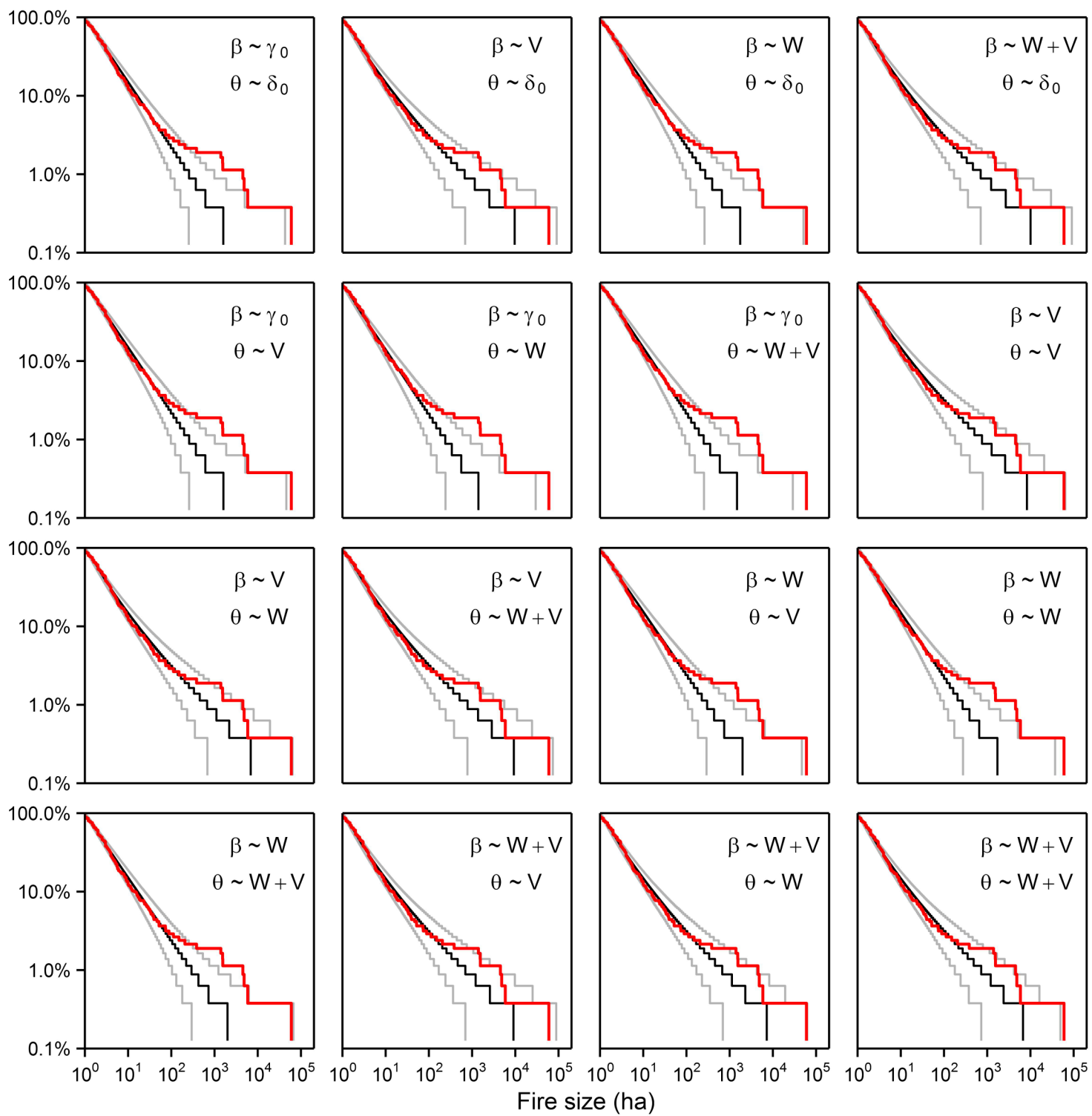

Supplement: S7 Fig — β0 and γ0, intercepts; W, fire weather and V, land-cover terms. (PDF) [file pone.0179294.s007.pdf]

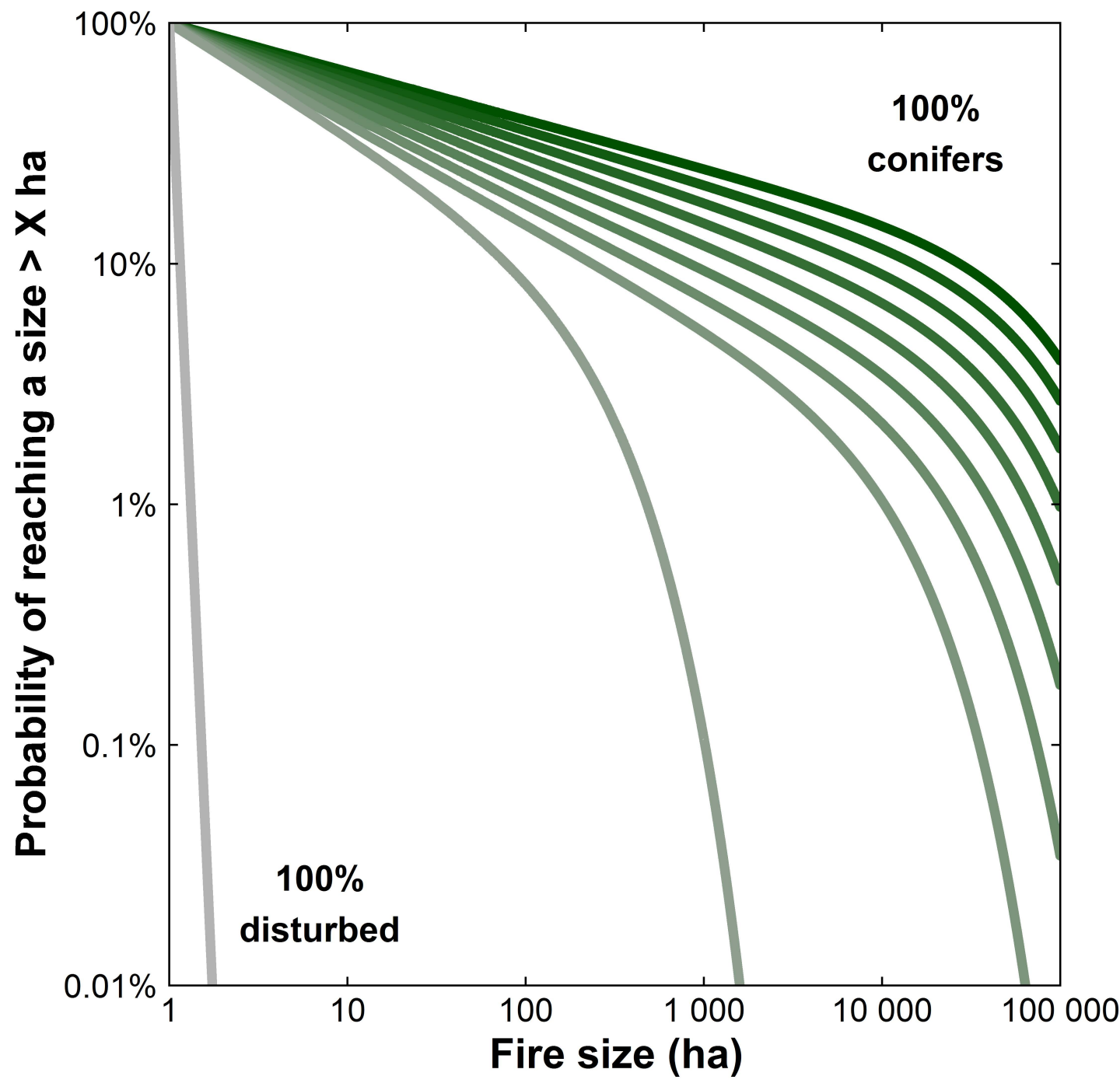

Supplement: S8 Fig — Predicted FSDs for hypothetical landscapes from 100% conifer to 100% disturbed in 10% increments, under means of fire weather covariates. (PDF) [file pone.0179294.s008.pdf]
