# Supplementary material for: Land cover, more than monthly fire weather, drives fire-size distribution in Southern Québec forests: Implications for fire risk management
Source: PLoS One. 2017 Jun 13;12(6):e0179294. doi: 10.1371/journal.pone.0179294 (PMC5469487; doi:10.1371/journal.pone.0179294)
Supplement: S1 Table — HW, hardwood; CN, coniferous; D, recently disturbed; O, open areas; WT, open water. (PDF) [file pone.0179294.s009.pdf]

		HW	CN	D	O	WT	MDC			
							May	June	MayJune	MayJuly
	**HW**	1.00								
	**CN**	-0.79	1.00							
	**D**	-0.58	0.32	1.00						
	**O**	-0.18	-0.13	-0.20	1.00					
	**WT**	-0.09	-0.19	-0.02	-0.29	1.00				
**MDC**	**May**	-0.10	0.09	0.03	-0.05	0.11	1.00			
	**June**	-0.22	0.16	0.12	0.01	0.08	0.59	1.00		
	**MayJune**	-0.20	0.15	0.09	-0.01	0.10	0.83	0.94	1.00	
	**MayJuly**	-0.18	0.14	0.06	-0.02	0.14	0.61	0.63	0.69	1.00
